# The diagnostic value of artificial intelligence in differentiating follicular thyroid cancer from follicular thyroid adenoma: A meta-analysis

**DOI:** 10.1097/MD.0000000000044745

**Published:** 2025-10-03

**Authors:** Di Wu, Chengfei Sun, Yilin Hou, Jiayue Sun, Xiyu Zhang, Yunfei Zhang

**Affiliations:** a Department of Ultrasound, The First Hospital of China Medical University, Shenyang, China; b Department of Ultrasound, General Hospital of Fushun Mining Bureau of Liaoning Health Industry Group, Shenyang, China; c Class 3, Grade 2023, Science High School, Northeast Yucai School, Shenyang, China.

**Keywords:** artificial intelligence, follicular thyroid adenoma, follicular thyroid carcinoma, machine learning, meta-analysis

## Abstract

**Background:**

Follicular thyroid carcinoma (FTC) is the second most common thyroid malignancy but is challenging to preoperatively distinguish from follicular adenoma. Artificial intelligence (AI) has emerged as an auxiliary diagnostic tool, yet published studies show variable performance. This meta-analysis aims to evaluate the overall diagnostic accuracy of AI in differentiating FTC from benign lesions.

**Methods:**

Literature searches were independently conducted across the PubMed, Embase & Medline (via Embase.com), Web of Science, Cochrane Library, and Ovid English medical databases. The diagnostic accuracy of AI was compared against the reference standard of histopathology. Pooled sensitivity, specificity, diagnostic odds ratio and area under the curve were calculated to assess AI accuracy. Meta-regression analyses were performed to investigate heterogeneity related to test set size, validation strategy, and machine learning model type.

**Results:**

We analyzed a total of 7 studies involving 3163 follicular thyroid neoplasms (comprising 1876 follicular thyroid adenomas [FTAs] and 1287 FTCs). The pooled sensitivity and specificity of AI for differentiating FTC from FTA were 0.73 (95% CI: 0.70–0.75) and 0.87 (95% CI: 0.86–0.89), respectively. The pooled positive and negative likelihood ratios were 6.19 (95% CI: 3.92–9.79) and 0.28 (95% CI: 0.17–0.46). The diagnostic odds ratio was 22.81 (95% CI: 10.17–51.16), and the area under the curve was 0.94. Meta-regression results indicated no significant heterogeneity associated with validation strategy (*P* = .25). However, test set size (*P* = .02) and publication year (*P* = .04) were identified as potential significant sources of heterogeneity. Subgroup analyses revealed that studies with a test set size > 1000 cases demonstrated superior accuracy compared to those with <1000 cases. Regarding validation strategy, studies utilizing cross-validation yielded better performance than those using holdout validation.

**Conclusion:**

Overall, AI demonstrates promising diagnostic utility in differentiating FTC and FTA. Studies employing larger test sets (>1000 cases) achieved higher accuracy than those with smaller test sets (<1000 cases). Furthermore, validation using cross-validation strategies outperformed non-cross-validation (holdout) approaches.

## 1. Introduction

FTC exhibits a relatively high incidence rate, second only to papillary thyroid carcinoma, accounting for approximately 10% to 20% of all thyroid malignancies.^[1]^ Although less common than papillary thyroid cancer, follicular thyroid cancer presents more frequently with distant metastases via hematogenous spread and is associated with higher mortality.^[2–4]^ Consequently, early identification of FTC holds significant clinical importance. Follicular thyroid neoplasms are primarily classified into FTC and FTA.

Current routine preoperative diagnostic methods rely mainly on ultrasonographic features and fine needle aspiration cytology. While ultrasound can assess characteristics such as nodule size, shape, echogenicity, margin features, calcification patterns, and vascularity, distinguishing between FTC and FTA preoperatively remains substantially challenging.

Fine needle aspiration cytology serves as a crucial method for differentiating benign from malignant thyroid nodules. However, FTC and FTA exhibit extreme cytomorphological similarities, presenting significant challenges in providing definitive pathological diagnoses.^[5,6]^ Currently, definitive differentiation still requires postoperative pathological examination following thyroidectomy, which relies on identifying capsular or vascular invasion surrounding the lesion.^[7]^

In recent years, artificial intelligence (AI) has rapidly evolved as an innovative tool and gained widespread application across various medical specialties.^[8]^ As a noninvasive approach, AI can reveal unique tumor characteristics from medical images. Multiple computational methods and models capable of extracting meaningful features from medical images offer clinicians novel diagnostic tools, potentially reducing the need for invasive procedures. Numerous studies investigating the role of AI methods in differentiating follicular thyroid neoplasms have been published in recent years. However, the reported results show considerable variation, with sensitivities ranging from 32% to 96% and specificities from 74% to 96%.^[9–15]^ Therefore, this study aims to evaluate the diagnostic performance of AI in differentiating follicular thyroid neoplasms through a meta-analysis.

## 2. Materials and methods

### 2.1. Literature search

This study complied with PRISMA recommendations.^[16,17]^ Independent literature searches of English medical databases including PubMed, Embase and Medline (via Embase.com), Web of Science, Cochrane Library, and Ovid were conducted to identify studies evaluating the differential diagnosis of follicular thyroid tumors. The search strategy is detailed in Table 1. Duplicate articles were manually excluded. Relevant unpublished materials were considered, but no suitable studies meeting inclusion criteria were identified. Two independent researchers performed the literature search, which was updated through September 30, 2024, with no start date restrictions.

**Table 1:** Search strategy of each database.

Database	Strategy
PubMed	(((“Adenocarcinomas Follicular”[Mesh]) OR Follicular Thyroid Carcinoma)) AND (((“Adenoma”[Mesh]) OR Adenomas Follicular)) AND (((((((((“Artificial Intelligence”[Mesh]) OR Computational Intelligence) OR Computer Reasoning) OR Knowledge Representation (Computer)) OR Machine Intelligence) OR Computer aided diagnosis) OR Machine learning) OR “Radiomics”[Mesh]))
Embase and Medline (Embase.com)	(#1) follicular AND carcinoma OR (follicular AND cancer) (#2) thyroid AND adenoma OR (autonomous AND thyroid AND adenoma) OR (thyroid AND gland AND adenoma) (#3) machine AND intelligence OR (artificial AND intelligence) OR (computer AND aided AND diagnosis) OR (machine AND learning) OR (radiomics) (#4) #1 AND #2 AND #3
Cochrane Library	(#1) MeSH descriptor: [Adenocarcinoma Follicular] explode all trees (#2)(Adenocarcinoma Follicular) OR (Follicular Thyroid Carcinoma) (Word variations have been searched) (#3)#1 OR #2 (#4)MeSH descriptor: [Adenoma] explode all trees (#5)(Adenoma) OR (Adenomas Follicular) (Word variations have been searched) (#6)#4 OR #5 (#7)MeSH descriptor: [Artificial Intelligence] explode all trees (#8)(Artificial Intelligence) OR (Computational Intelligence) OR (Computer Reasoning) OR (Computer aided diagnosis) OR (machine learning) (Word variations have been searched) (#9)MeSH descriptor: [Radiomics] explode all trees (#10)#7 OR #8 OR #9 (#11)#3 AND #6 AND #10
Web of Science	TOPIC: ((Adenocarcinomas Follicular) OR (Follicular Thyroid Carcinoma)) AND TOPIC: ((Adenoma) OR (Adenomas Follicular)) AND TOPIC: ((Artificial Intelligence) OR (Computational Intelligence) OR (Computer Reasoning) OR (Machine Intelligence) OR (Computer aided diagnosis) OR (Machine learning) OR (Radiomics))
Ovid	(#1)(Adenocarcinomas Follicular OR Follicular Thyroid Carcinoma).af. (#2) (Adenoma OR Follicular Adenomas).af. (#3)(Artificial Intelligence OR Computational Intelligence OR Computer Reasoning OR Machine Intelligence OR Computer aided diagnosis OR Machine learning OR Radiomics).af. (#4)#1 and #2 and #3

OR = odds ratio.

### 2.2. Inclusion and exclusion criteria

All articles were independently assessed by 2 investigators.

Inclusion criteria:

1. Studies approved by an ethics committee or institutional review board
2. Evaluation of AI’s diagnostic value for differentiating follicular thyroid tumors
3. Postoperative histopathology as reference standard
4. Complete data for calculating true positive, false positive, false negative, and true negative cases

Exclusion criteria:

1. Non-English publications including reviews, case reports, letters, conference reports, editorials
2. Studies with insufficient data after contacting corresponding authors via email (excluded if no response within 15 days)
3. When multiple studies originated from the same department, those with older publication dates or smaller sample sizes were excluded

All disagreements were resolved through consensus.

### 2.3. Data extraction

Data extraction was performed independently by 2 investigators. Extracted information included: first author, country, publication year, patient age, sex distribution, number of patients, number of lesions, reference standard, lesion type, methodology, and counts of true positive, false positive, false negative, and true negative. When authors did not explicitly report diagnostic thresholds, these were defined using the Youden index method. Disagreements were resolved through consensus.

### 2.4. Quality assessment

The Quality Assessment of Diagnostic Accuracy Studies (QUADAS-2) tool recommended by the UK National Institute for Health and Care Excellence was used to evaluate study quality.^[18]^ QUADAS-2 focuses on evaluating clinical applicability and assessing risk of bias. Two investigators independently completed bias risk assessment, categorizing responses as “yes,” “no,” or “unclear” for key questions in each domain, and rated overall bias risk as “low,” “high,” or “unclear.” Disagreements were resolved through discussion.

### 2.5. Statistical analysis

Analyses employed Meta-Disc 1.4, STATA 14.0, RevMan 5.3, and SPSS 26.0 (IBM Corp., Armonk). Spearman’ s correlation coefficient assessed threshold effects. Heterogeneity was evaluated using Cochran’s *Q* statistic and *I*² tests. A random-effects model was applied when heterogeneity *P*-value < .05 or *I*² ≥ 50%; otherwise, a fixed-effects model was used. Meta-Disc calculated pooled sensitivity, specificity, diagnostic odds ratio (DOR), area under the curve (AUC), and Q* index. Meta-regression explored potential heterogeneity sources. RevMan software facilitated literature quality assessment. Deeks’ funnel plots generated in STATA assessed publication bias (*P* < .05 indicating significance). Cohen’ s κ analysis in SPSS evaluated interobserver agreement during article screening and QUADAS application.

## 3. Results

### 3.1. Literature search and characteristics of included studies

**Figure 1.**
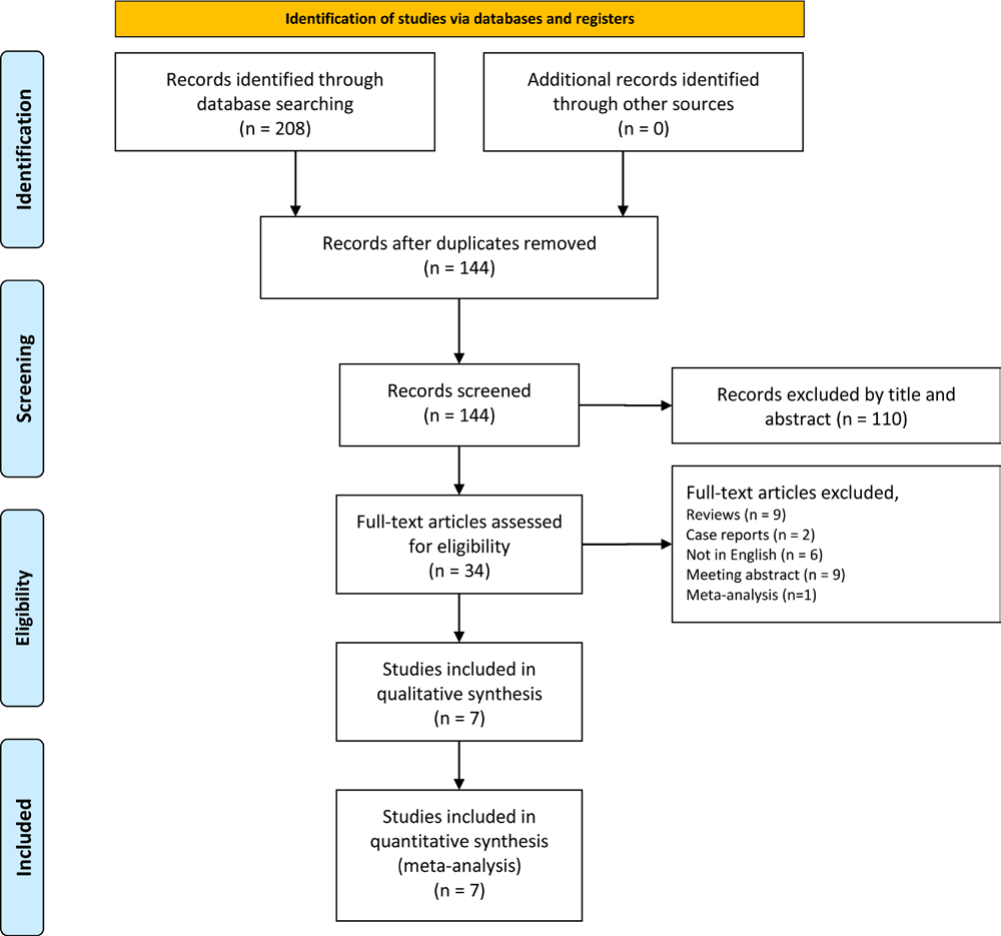
The research selection and literature retrieval process of this paper.

Seven studies published between 2017 and 2024 were included in the meta-analysis, comprising 3163 follicular thyroid lesions^[9–15]^ (Fig. 1). Key characteristics of the included studies are summarized in Table 2. Disagreements arose between reviewers during title and abstract screening. However, interobserver agreement was substantial (κ = 0.86; 95% CI: 0.72–0.99). All disputed articles were retained at this stage. Subsequent screening steps showed perfect agreement (κ = 1).

**Table 2:** Main characteristics of included studies.

	Author	Country	Year	Age	Male/female/unknown	Number of patients	Number of lesions	Reference standard
1	Chen, Weiwei et al	China	2024	–	–	279	279	Postoperative pathology
2	Yu, Bing et al	China	2022	42.8	43/86/0	129	129	Postoperative pathology
3	Xu, Dong et al	China	2022	–	–	607	699	Postoperative pathology
4	Yang, Bailin et al	China	2020	–	–	300 (images)	300	Postoperative pathology
5	Seo, Jeong-Kweon et al	Koera	2017	47.2	73/250/10	333	307	Postoperative pathology
6	Yang, Zheyu et al	China	2023	44.3	118/234/0	352	1392	Postoperative pathology
7	Shin, Iiah et al	Koera	2020	47.2	79/261/0	340	348	Postoperative pathology
	Type of lesions (number of lesions)		Identification method		TP	FP	FN	TN
1	Follicular thyroid carcinoma (n = 86) follicular thyroid adenoma (n = 193)		Neural network based on multiscale feature fusion		68	19	18	174
2	Follicular thyroid carcinoma (n = 28) follicular thyroid adenoma (n = 101)		Combined ultrasound radionics features and clinical ultrasound features		21	13	7	88
3	Follicular thyroid carcinoma (n = 167) follicular thyroid adenoma (n = 241) adenomatoid hyperplasia nodule n = 291)		Artificial intelligence ultrasound system		115	63	52	178
4	Follicular thyroid carcinoma (n = 146) follicular thyroid adenoma (n = 154)		Cascaded convolutional neural network		140	6	6	148
5	Follicular thyroid carcinoma (n = 77) follicular thyroid adenoma (n = 230)		Convolutional Neural Network		55	16	22	214
6	Follicular thyroid carcinoma (n = 687) follicular thyroid adenoma (n = 705)		A deep learning model (FThyNet)		506	99	181	606
7	Follicular thyroid carcinoma (n = 96) follicular thyroid adenoma (n = 252)		An artificial neural network (ANN)		31	25	65	227

– Indicates not mentioned in the article.

ANN = artificial neural network , FN = false negative, FP = false positive, TN = true negative, TP = true positive.

### 3.2. Quality assessment

**Figure 2.**
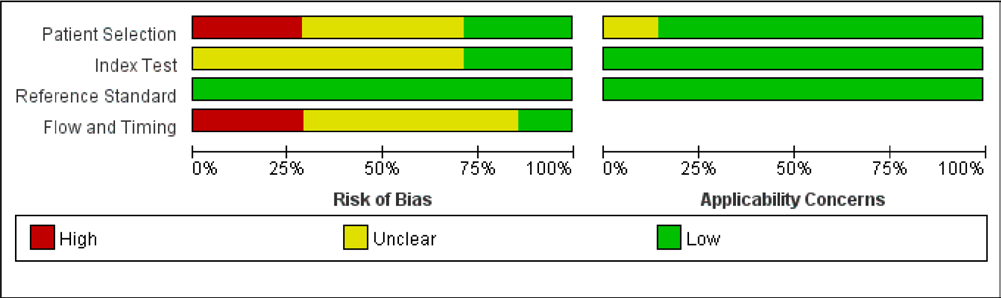
Quality assessment of the included studies using the “QUADAS” questionnaire. QUADAS = Quality Assessment of Diagnostic Accuracy Studies.

Figure 2 presents the quality assessment outcomes. Most criteria were appropriately addressed, indicating high overall study quality. Inter-reviewer agreement was excellent (κ = 0.816).

### 3.3. Diagnostic accuracy for FTC versus FTA differentiation

**Figure 3.**
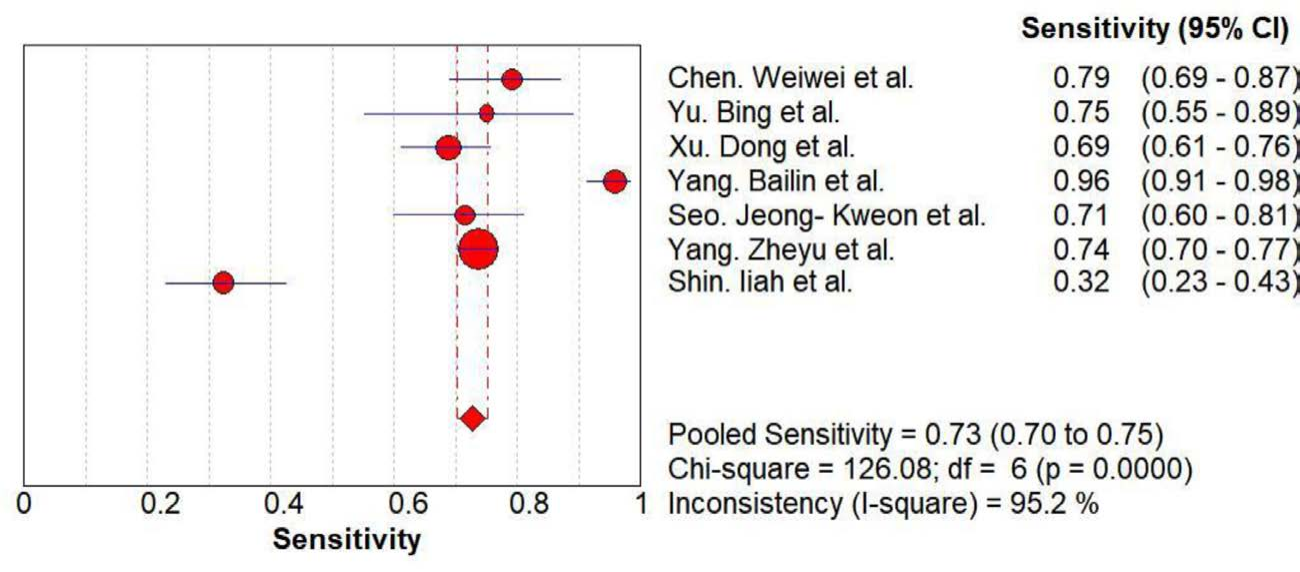
The Forest map results of sensitivity. CI = confidence interval.

**Figure 4.**
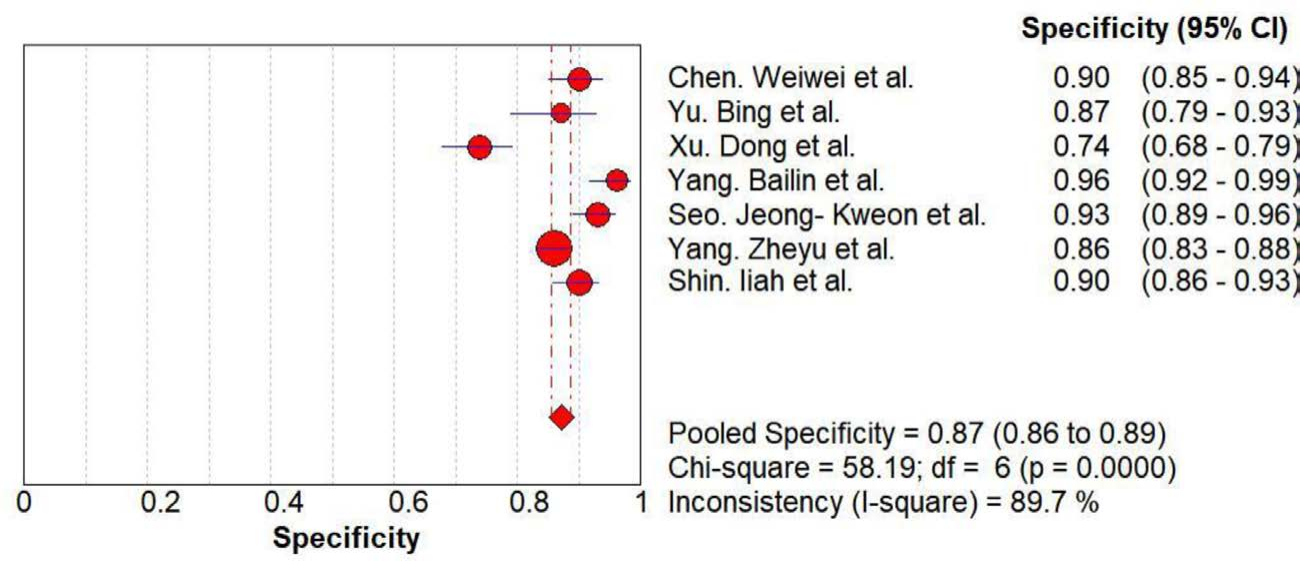
The Forest map results of specificity. CI = confidence interval.

**Figure 5.**
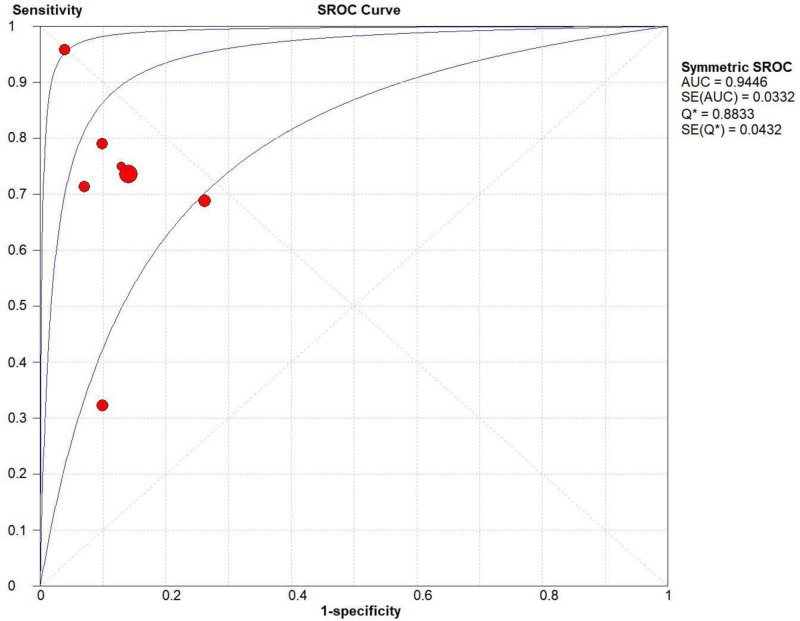
The summary receiver operating characteristic (SROC) curve of their diagnostic accuracy obtained in this paper. AUC = area under the curve, ESS = effective sample size, SE = standard error, SROC = the summary receiver operating characteristic curve.

Threshold effect analysis revealed no heterogeneity (Spearman’s correlation coefficient = −0.500, *P* = .253). The diagnostic accuracy of AI in differentiating FTC from FTA demonstrated pooled sensitivity of 0.73 (95% CI: 0.70–0.75), specificity of 0.87 (95% CI: 0.86–0.89), and DOR of 22.81 (95% CI: 10.17–51.16). The summary receiver operating characteristic curve indicated high overall accuracy with an AUC of 0.94 (Q* = 0.88; Figs. 3–5).

### 3.4. Heterogeneity analysis

Significant heterogeneity was detected by Cochran’s *Q* test (*P* < .001) and *I*² test (*I*² = 92.7%). Meta-regression exploring non-threshold heterogeneity sources evaluated test set size (group 1: >1000 cases; group 2: <1000 cases), validation strategy (group 1: cross-validation; group 2: non-cross-validation), and publication year (group 1: 2020 publications; group 2: non-2020 publications). Validation strategy showed no significant association with heterogeneity (*P* = .25). However, test set size (*P* = .02) and publication year (*P* = .04) were significant heterogeneity sources. Studies with >1000 test cases demonstrated superior accuracy compared to those with <1000 cases. Cross-validation outperformed non-cross-validation approaches (Table 3).

**Table 3:** Results of the meta-regression and subgroup analysis.

Subgroup	Number of studies	Pooled sensitivity (95% CI)	Pooled specificity (95% CI)	Pooled DOR (95% CI)	AUC	*P*-value
Test set size						.023
>1000 cases	1	0.96 (0.90–0.97)	0.96 (0.91–0.97)	575.01 (183.23–1817.45)	0.98	
<1000 cases	6	0.70 (0.67–0.72)	0.86 (0.86–0.88)	14.37 (7.53–27.43)	0.89	
Validation strategies						.250
Cross-validation	4	0.73 (0.68–0.78)	0.91 (0.89–0.93)	34.91 (5.46–223.15)	0.98	
Non-cross-validation	3	0.73 (0.70–0.76)	0.85 (0.83–0.87)	14.79 (6.45–33.88)	0.76	
Year of publication						.041
2020	2	0.71 (0.65–0.76)	0.92 (0.89–0.95)	48.70 (0.36–6570.70)	–	
Non-2020	5	0.73 (0.70–0.76)	0.86 (0.84–0.88)	18.22 (9.68–34.30)	0.77	

AUC = area under the curve, DOR = diagnostic odds ratio.

### 3.5. Publication bias

**Figure 6.**
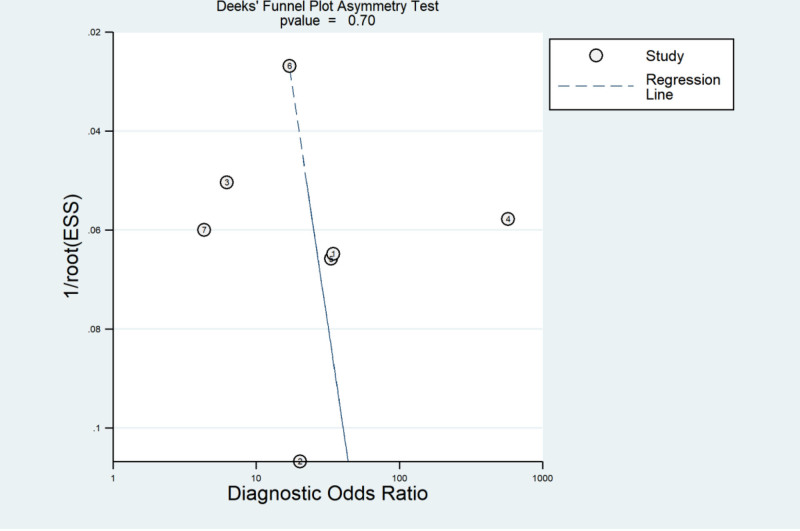
The results of Deek’s funnel plot: no significant bias was found.

Deeks’ funnel plot analysis revealed symmetrical scatter distribution around the regression line (*P* = .70, *P* > .05), indicating no significant publication bias (Fig. 6).

## 4. Discussion

FTC, the second most common differentiated thyroid cancer originating from follicular epithelium, has been reported to exhibit a rising incidence.^[19]^ Early detection and diagnosis are therefore crucial for clinical management and prognosis. Ultrasound remains the primary imaging modality for thyroid disorders. Recent advancements in ultrasound technology and the introduction of C-TIRADS and ACR-TIRADS guidelines have improved diagnostic accuracy for malignancies, particularly papillary thyroid carcinoma.^[20]^ However, distinguishing FTC solely by ultrasound remains challenging, necessitating novel diagnostic approaches.

AI has emerged as a research focus in thyroid tumor diagnosis,^[21]^ demonstrating diagnostic capabilities that may surpass those of experienced clinicians.^[15]^ Over 100 AI and machine learning-enabled medical devices are currently under FDA review.^[22]^ While preliminary progress has been made in differentiating FTC from FTA using AI, significant variability exists across studies due to differences in machine learning models, sample sizes, validation strategies, and segmentation precision. For instance, Yang et al^[13]^ achieved 96.00% accuracy using a cascaded learning architecture combining prior-based level sets and deep convolutional neural networks, whereas Xu et al^[12]^ reported only 71% accuracy with an unmodified thyroid nodule diagnosis software development kit. These discrepancies highlight the need for further validation of AI’s diagnostic value, prompting this systematic evaluation via meta-analysis.

Our analysis included 7 studies encompassing 3163 lesions (1876 FTAs, 1287 FTCs; malignancy rate 40.7%). The pooled sensitivity was 0.73 (95% CI: 0.70–0.75), specificity was 0.87 (95% CI: 0.86–0.89), positive likelihood ratio was 6.19 (95% CI: 3.92–9.79), negative likelihood ratio was 0.28 (95% CI 0.17–0.46), diagnostic DOR was 22.81 (95% CI: 10.17–51.16), and AUC was 0.94. These results indicate robust diagnostic performance with high specificity and clinical utility. However, the moderate sensitivity aligns with Yang et al’s findings,^[14]^ potentially attributable to limited FTC samples for model training and AI’s focus on textural features at nodule margins. While benign nodules typically exhibit regular textures and malignancies irregular patterns, some FTCs present ambiguous features.^[23]^

Heterogeneity testing in this study yielded *Q* = 82.48 and *I*² = 92.7%, suggesting significant heterogeneity. The Spearman correlation coefficient of −0.500 with *P* = .253 (*P* > .05) indicates no threshold effect. Meta-regression revealed no heterogeneity associated with validation strategy (*P* = .25). Meta-regression analyses demonstrated by the number of test sets (*P* = .02) as well as year of publication (*P* = .04). Subgroup analyses demonstrated: larger test sets (>1000 cases) outperformed smaller cohorts in sensitivity (0.96 vs 0.70), specificity (0.96 vs 0.86), and AUC (0.98 vs 0.89). Cross-validation yielded higher specificity (0.91 vs 0.85) and DOR (34.91 vs 14.79). Studies published in 2020 showed higher sensitivity (0.71 vs 0.73) and specificity (0.92 vs 0.86). Interpretation requires caution due to residual within-group heterogeneity, necessitating further high-quality studies.

Study limitations include: Restriction to English-language publications may introduce language bias. Inclusion of retrospective studies only, with limited multicenter data. Geographic diversity potentially causing selection bias. Small subgroup sizes limiting analytical power.

In conclusion, AI demonstrates high specificity (0.87) and AUC (0.94) for differentiating FTC from FTA, with moderate sensitivity (0.73) suggesting substantial clinical value. Given limitations in study quantity and quality, further high-quality prospective studies are warranted to validate these findings.

## Acknowledgments

Thanks for the guidance of Prof. Yun-fei Zhang and thanks for the cooperation of our team.

## Author contributions

**Conceptualization:** Xiyu Zhang.

**Data curation:** Xiyu Zhang.

**Formal analysis:** Xiyu Zhang.

**Investigation:** Yilin Hou, Jiayue Sun.

**Methodology:** Yilin Hou, Jiayue Sun.

**Project administration:** Yilin Hou.

**Resources:** Chengfei Sun.

**Software:** Chengfei Sun.

**Supervision:** Chengfei Sun.

**Validation:** Chengfei Sun

**Visualization:** Di Wu.

**Writing – original draft:** Di Wu.

**Writing – review & editing:** Di Wu, Yunfei Zhang.
